# Supplementary material for: Operating bedside cardiac ultrasound program in emergency medicine residency: A retrospective observation study from the perspective of performance improvement
Source: PLoS One. 2021 Apr 2;16(4):e0248710. doi: 10.1371/journal.pone.0248710 (PMC8018668; doi:10.1371/journal.pone.0248710)
Supplement: Attachment — Submitted filename: Answer to comment.docx [file pone.0248710.s002.docx]

**Author’s reply to reviewers' comments:**

On behalf of authors, thank you for the very valuable comments by the reviewer on our paper. We have attempted to address every point commented on by the reviewer in the revised manuscript. While we believe that we have addressed all of the reviewer’s concerns, we would be more than pleased to write additional revisions if needed.

We highlighted all changes in red. Author’s answers or explanations are in blue.

Correspondent author

Jae Yun Jung, MD

Title: Operating bedside cardiac ultrasound program in emergency medicine residency: a retrospective observation study from the perspective of performance improvement
Journal: Plos one

Reviewer #1: Thanks for the opportunity to review this.

As you say, it shows objective improvement in competence and subjective confidence following a structured POCUS training programme.

I have a couple of queries

- why does the mean time in program (3 months) and the mean period between early and late phases (20 weeks) not match? You state early and late phase are at the beginning and end of the programme?

(ANSWER) Thank you for the review. Residents take practice rotations, which include dispatch to other hospitals, on a monthly basis, so the PoCUS rotation duty not the same for each individual. The gap between first and last rotation differ for individuals, which is demonstrated as mean period between phases. We described it more clearly in manuscript according to your review.

(REVISION: Results) The mean period between the early and late phases, which is almost same as the gap between first and last rotation, was 20 weeks. The mean participation in the PoCUS program was 3 months, which was not the same for each individual due to rotation schedule including dispatching to other hospitals.

- I would value some more detail / clarity about the qualifications or experience of the faculty / assessors. it is not clear how experienced or suitable they are at assessing the quality of images (I am sure they are perfectly qualified but to allow reproducibility we need more details of their qualifications)

(ANSWER) Thank you for the review. We added detail explanation of experience of the faculty in the manuscript accordingly.

(REVISION: Methods) Two independent emergency specialists were consulted to evaluate the cases. They are PoCUS faculty members who have practical experience with more than 200 cardiac PoCUS and work as PoCUS instructors for emergency physician and medical school student.

Reviewer #2: The article is an interesting analysis on the Operating bedside cardiac ultrasound program in emergency medicine residency. The article is clear and the main results well represented. To make the article more appealing for readers would be of interest to know also attitude in the use of color doppler and pulse wave doppler for assessment of diastolic function and have a dedicated issue on assessment of valve disease.

(ANSWER) Thank you for the review and valuable comment. We sincerely considered to include performance and attitude toward assessment of valve function or diastolic function in this study. Since near half of participants were under YOG 2, for whom comprehensive cardiac ultrasound is not the requirement, we excluded them. Because it remains significant limitation, we added it in the manuscript.

(REVISION: Limitations) Next, concrete description or analysis for diastolic function and valve function was not conducted. Though some description for them was in interpretation, we considered competency of participants is insufficient, which including diastolic function and valve function seems inappropriate.

Reviewer #3: Thank you for the opportunity to review this manuscript on a retrospective analysis of the impact of a dedicated POCUS on attitudes and output of a POCUS program in an EM residency. Overall i found the data contained in the manuscript to be informative and very useful for readers who play a role in an EM based POCUS program. Strengths of the manuscript include:

-clearly stated statement of objective

-an detailed enough description of what was done that a similar researcher could reproduce this study in their own EM program

-utilizing independent research coordinators to randomly select the cases from the two periods that were evaluated by the study team

-detailed explanation of the items that were evaluated in each of the cardiac ultrasound views

-employing a sensitivity analysis

The following items are things that i believe should be addressed to improve the manuscript:

-at the end of the 1st paragraph in the Introduction you should state that the ACGME RRC EM requires/mandates training in POCUS and cite their documents. it would be a more powerful and accurate statement to highlight the importance of POCUS for the general readership

(ANSWER) Thank you for the review. We added as your recommendation in Introduction section

(REVISION: Introduction) In the United States, PoCUS has been requirement of residency training in emergency medicine (1).

-although the objective statement at the conclusion of the Intro is explicitly stated, i do take issue with the use of the term "perception". It is not clearly defined anywhere in the manuscript and b/c you are including it in your objective statement i think you should either revise the verbiage of the objective statement, or include in the methods section how you intend to measure "perception"

(ANSWER) Thank you for the review. We changed the objective statement accordingly to be consistent with the whole manuscript.

(REVISION: Introduction) The secondary outcome was a change in attitude and confidence toward PoCUS in emergency practice.

-the use of the term "screening" in the Methods section seems misplaced. It almost seems like you simply excluded residents who didn't follow the parameters of the POCUS rotation, rather than screening. I think you should eliminate the use of the word screening here and simply describe precisely how you went about selecting the participants

(ANSWER) Thank you for the review. We changed the wording as your recommend

(REVISION: Methods) Emergency medicine residents who followed emergency bedside rotation duties during the study period, were enrolled.

-your scoring system, although appearing to be quite robust, is certainly novel. Did you do anything to internally validate this measure?

(ANSWER) Thank you for the review and valuable comment. This scoring system has been developed for the first time, and it does not used in any other study yet. We plan to use it for future research. Though it was demonstrated as consistent between assessors, it is still significant limitation, which we added it in Discussion section.

(REVISION: Discussion) Next, scoring system used in this study has not been validated for various environment. Though it was developed based on the standard guidelines, further research for validation is needed.

-going back to the "screening" issue, by my interpretation of the manuscript there were 33 eligible participants amongst the 2nd-4th year residents, and ultimately 22 of them were included b/c 6 refused to complete the questionnaire and 5 were excluded b/c they didn't perform or document appropriately their POCUS exams. If this is indeed true, i think a flow diagram outlining this would be a much better way to describe this then the way it is currently described in the manuscript using the "screening" terminology

(ANSWER) Thank you for the review. Actually, among total 28 eligible participants, 5 were excluded due to inappropriate examination data, and among them, 6 refused to complete the questionnaire. As a result, examinations of 23 participants were analyzed for performance and questionnaire of 22 participants for attitude. We clarified the flow in Result section more clearly, as your comment.

(REVISION: Result) From April 2018 to February 2019, a total of 28 emergency medicine residents participated in the PoCUS program. 5 participants were excluded from the performance analysis due to the omission of image data or interpretations. Survey analysis was conducted with 22 residents since a participant refused to complete the questionnaire among them. The demographics and characteristics of the study participants are described in Table 3.

- on lines 229-230 of the Results section you state that younger age had a negative association with performance improvement, however on lines 243-244 of the Discussion section you state that younger age had an association with marked improvement. Please resolve this discrepancy.

(ANSWER) Thank you for the review. In multivariable linear regression analysis model for performance improvement, age showed negative beta coefficient. We checked the manuscript and revised accordingly.

(REVISION: Result) In multivariable linear regression analysis, we found that age, early-phase score and prior high confidence had a negative association with performance improvement.

-since this study was essentially only looking at cardiac POCUS, i would recommend rewording much of the paragraph on lines 263-276

(ANSWER) Thank you for the review. We reviewed the section and revised the wording as you recommended.

(REVISION: Discussion) The results of this research demonstrated an effective way to increase the performance and favorable attitudes of cardiac PoCUS in residents. Emergency medicine residents struggle in overcrowded emergency departments to manage various patients with severe needs, which results in a stressed and dissatisfied training period. It often leads residents to overload themselves to complete the mandatory cardiac PoCUS training sessions off duty. On the other hand, procedural training without real clinical practice can blur the effect of education programs. Furthermore, a recent study showed that doctors recognize their need for sufficient practice with certain procedures to build competency. We can infer that less education and more practice would help improve both performance and attitude on cardiac PoCUS.

-lines 277-283 seem to be discussing practice of POCUS that occurs "on shift" in an ED, although this is not explicitly stated, i would rephrase to include this term if this is indeed what you are discussing here

(ANSWER) Thank you for the review. We would like to discuss about the routine application of cardiac PoCUS on specific population. The novel practice protocol with cardiac PoCUS may have favorable effect on clinical course. We reworded the phrase to clarify the objectives of discussion.

(REVISION: Discussion) Our research focused on performance improvement of cardiac PoCUS with real practice, but the clinical benefit has not been well studied. We are planning an additional study to evaluate the association between the implementation of cardiac PoCUS protocol for specific population and improvement of clinical outcomes.

-Line 300 referring to the lack of a cardiologist performed evaluation is not a Limitation. Cardiologists are not content experts in cardiac POCUS and need not be the ones performing evaluations of POCUS exams in the ED

(ANSWER) Thank you for the review and valuable comment. We revised the detail explanation of limitation in terms of assessor certification which is inconsistent with previous studies on PoCUS.

(REVISION: Discussion) Fourth, evaluation of performance was conducted by emergency medicine specialists. In United States, certification for PoCUS competency in emergency practice has been developed for various institutes. We consider certification of assessor as appropriate with more than 200 cardiac PoCUS experienced, and qualification for PoCUS instructor in Seoul National University Hospital Department of Emergency Medicine. Detailed components to prove the practitioner's credentials may not match perfectly due to lack of official PoCUS certificate programs in South Korea, which can be another limitation.

1.	Heller MB, Mandavia D, Tayal VS, Cardenas EE, Lambert MJ, Mateer J, et al. Residency training in emergency ultrasound: fulfilling the mandate. Acad Emerg Med. 2002;9(8):835-9.
