# Supplementary material for: Operating bedside cardiac ultrasound program in emergency medicine residency: A retrospective observation study from the perspective of performance improvement
Source: PLoS One. 2021 Apr 2;16(4):e0248710. doi: 10.1371/journal.pone.0248710 (PMC8018668; doi:10.1371/journal.pone.0248710)
Supplement: Attachment — Submitted filename: Answer to comment.docx [file pone.0248710.s003.docx]

**Author’s reply to reviewers' comments:**

On behalf of authors, thank you for the very valuable comments by the reviewer on our paper. We have attempted to address every point commented on by the reviewer in the revised manuscript. While we believe that we have addressed all of the reviewer’s concerns, we would be more than pleased to write additional revisions if needed.

We highlighted all changes in red. Author’s answers or explanations are in blue.

Correspondent author

Jae Yun Jung, MD

Title: Operating bedside cardiac ultrasound program in emergency medicine residency: a retrospective observation study from the perspective of performance improvement
Journal: Plos one

Reviewer #2:

Two specific areas of the revised manuscript In the Methods section, lines 91-93. How did you treat the scenario where a resident saved some of the images of the exams they did, but not all of them? Were they excluded, or did you only exclude residents who did not save any of their images? Selection bias is possible (likely) given the manner in which most residents save some, but not all of their images from exams they do on ultrasound rotations.

(ANSWER) Thank you for the review. Participants were excluded who did not saved the whole basic view of ultrasound. The entire set of basic view were introduced and educated to participants at the beginning of POCUS program. We considered that evaluating the best performance at each point is thought to be proper in comparing between phases. We clarified this at the manuscript more clearly.

(REVISION: Methods) Participants who did not save the whole basic views of ultrasound imaging in the picture archiving communication system or document their interpretations in medical records were excluded from the analysis.

(REVISION: Methods) All residents had to take a comprehensive ultrasound workshop for basic echocardiography, including basic views, by a cardiologist early in their 2nd years.

The other location in the manuscript where this comes up is in the Results section, lines 194-198. 35 pairs of questionnaires from 22 inhabitants means there was a lot of participants who only completed 1 survey…and also, the 22 participants is different from the 28 participants identified in the first sentence. What happened to the 6 residents w/o surveys? The next sentence says 5 were excluded so that still leaves you at 23 and not 22. The old manuscript says 6 participants refused to complete the questionnaire, why was this info excluded from the revision?

(ANSWER) Thank you for your valuable comments. First, 6 residents denied or neglect conducting surveys, as you indicated. 5 participants were excluded from performance analysis, not survey analysis. Total achieved questionnaires were 35 pairs, 1 pair for Early-phase and Late-phase. Which means there were 70 questionnaires for 22participants. There were 13 residents, who participated yearly POCUS program in succession (as described in manuscript, 2^nd^ ~ 4^th^ year of residents participate the program), conducted questionnaire in each year. We clarified this in the manuscript more clearly.

(REVISION: Results) From April 2018 to February 2019, a total of 28 emergency medicine residents participated in the PoCUS program. 46 ultrasound data from 23 participants were analyzed in performance evaluation, since 5 participants were excluded who did not save the whole basic ultrasound views or interpretations. In survey analysis, 6 participants refused to fill out questionnaire and 22 residents conducted 35 pairs (35 early-phase and 35 late phase) of questionnaires, since 13 residents participated yearly POCUS program in succession for study period.
